# Decreased PAX6 and DSG1 Protein Expression in Corneal Epithelium of Patients with Epithelial Basal Membrane Dystrophy, Salzmann Nodular Degeneration, and Pterygium

**DOI:** 10.3390/jcm14051456

**Published:** 2025-02-21

**Authors:** Tanja Stachon, Fabian N. Fries, Zhen Li, Loay Daas, Zoltán Zsolt Nagy, Berthold Seitz, Nóra Szentmáry

**Affiliations:** 1 Dr. Rolf M. Schwiete Center for Limbal Stem Cell and Aniridia Research, 66421 Homburg, Saarland, Germany; 2 Department of Ophthalmology, Saarland University Medical Center, 66421 Homburg, Saarland, Germanyberthold.seitz@uks.eu (B.S.); 3 Department of Ophthalmology, Semmelweis University, 1085 Budapest, Hungary

**Keywords:** epithelial basal membrane dystrophy, Salzmann’s nodular degeneration, pterygium, molecular markers

## Abstract

**Background/Objectives**: Evaluation of stem cell, keratin, retinoic acid metabolism markers and non-coding micro-RNAs (miRNAs) in conjunctival and corneal samples of patients with epithelial basal membrane dystrophy (EBMD), Salzmann nodular degeneration (SND), pterygium and congenital aniridia (CA), to detect similarities and differences in their pathogenesis. **Methods**: Impression cytology (IC) samples and corneal epithelial samples (CEs) of patients with EBMD, SND, pterygium, congenital aniridia, and healthy control subjects have been analyzed. The IC samples were subjected to qPCR, and the epithelial samples were subjected to qPCR and WB. Limbal epithelial stem cell markers, keratins, retinoic acid metabolism markers, and miRNAs were analyzed. **Results**: In conjunctival IC samples, PAX6 mRNA expression was significantly lower in EBMD, SND, pterygium, and CA compared to healthy controls (*p* ≤ 0.02). KRT13 mRNA expression was significantly higher in EBMD, SND, and pterygium (*p* ≤ 0.018), and FABP5 was increased in pterygium samples (*p* = 0.007). MiRNA-138-5p was significantly higher in aniridia samples than in normal controls (*p* = 0.037). In corneal epithelial samples, PAX6 protein, DSG1 mRNA and protein, miRNA-138-5p, and miR-204-5p expression were significantly lower in EBMD, SND, and pterygium samples than in controls (*p* ≤ 0.02). ALDHA1 mRNA expression was significantly lower (*p* < 0.0001), and FABP5 mRNA expression was significantly higher (*p* = 0.014) in pterygium samples than in controls. **Conclusions**: PAX6, DSG1, miR-138-5p, and miR-204-5p expression is decreased in the corneal epithelium of epithelial basal membrane dystrophy, Salzmann nodular degeneration, and pterygium subjects. In addition, there is a dysregulation of markers of the retinoic acid signaling pathway, such as ADH1A1 and FABP5, in the corneal epithelium of pterygium subjects. These changes may offer therapeutic targets in the treatment of these ocular surface diseases.

## 1. Introduction

**Figure 1:**
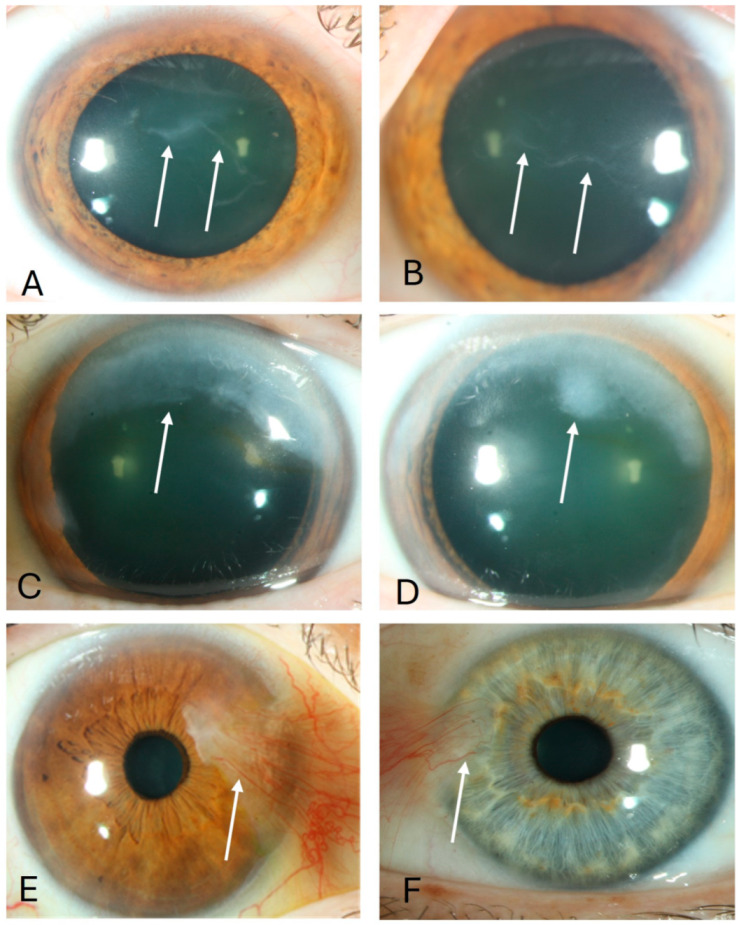
Epithelial basal membrane dystrophy ((**A**,**B**), arrows), Salzmann nodular degeneration ((**C**,**D**), arrows), and pterygium ((**E**,**F**), arrows) in our patients.

Epithelial basal membrane dystrophy (EBMD), Salzmann’s nodular degeneration (SND), and pterygium are all separate ocular surface disease entities; nevertheless, they are commonly present in combination [1,2,3] and are characterized by changes in the corneal epithelium, not rarely with subepithelial corneal scarring (Figure 1). Nevertheless, gene expression in corneal epithelial cells of EBMD, SND, and pterygium patients has not been described in detail. In congenital aniridia, with PAX6 haploinsufficiency in most of the cases, morphological changes of the corneal epithelium similar to EBMD, Salzmann nodules, and pterygium formation may all be observed [4].

EBMD affects approximately 2% of the population [5]. It is a genetically inherited disease with typical morphological changes of the corneal epithelium, either with map-, dot-, or fingerprint-like epithelial changes, which may result in recurrent corneal erosions and also visual impairment [5]. Although most cases are sporadic, EBMD may also be inherited in an autosomal dominant manner [6]. To our knowledge, in EBMD, dysfunction of the limbal stem cells has not been identified yet.

In SND, irregular whitish nodules appear under the corneal epithelium and above Bowman’s layer, but Bowman’s layer may also be usurated under the nodules. The nodules appear more often at the periphery than at the central cornea; nevertheless, they may increase in size and number with time [7]. SND usually appears in middle-aged patients and may also recur after removal, and its etiology still remains unclear. Some authors described its familial occurrence; some others assume an inflammatory origin. Some authors discuss whether external factors such as contact lens wear or previous corneal injuries may also promote SND development [8]. Nevertheless, dry eye disease or previous surgical interventions may also result in SND development [9,10]. Eberwein et al. suggested alterations of the limbal stem cells in SND [10].

In pterygium, there is focal, sectorial limbal stem cell insufficiency (LSZI), with conjunctivalization of the cornea. Genetic factors, chronic inflammation, and UV radiation may play a role in its development [11]. Due to the impaired limbal epithelial barrier, conjunctival epithelial cells migrate towards the corneal center. In recent years, the role of microRNAs has been discussed in pterygium pathogenesis [12], and some studies also performed transcriptome and proteomic profiles in tears of pterygium samples [11,13,14,15,16].

In contrast to EBMD, in SND and pterygium, there is a progressive dysfunction of the limbal stem cells. A progressive limbal stem cell deficiency is also observed in aniridia-associated keratopathy (AAK), which is additionally associated with increasing scarring and conjunctivalization of the cornea due to circular LSZI. In most patients with congenital aniridia, there is a PAX6 haploinsufficiency, which seems to be involved in dysfunction of the limbal epithelial stem cell niche and results in a decreased PAX6 mRNA and protein expression in conjunctival and corneal epithelial cells [17]. Several causes for the formation of a pterygium have already been discussed and summarized in the review by He [18].

LSZI and corneal surface irregularities are accompanied by ocular surface inflammation in most cases. Therefore, it is important to analyze gene expression changes at the conjunctival surface in these patients as well.

In EBMD, SND, and pterygium, significant changes in the epithelium become apparent as the conditions progress. This study analyzed PAX6 and TP63, two key transcriptional markers of corneal epithelial cells, along with desmoglein 1 (DSG1), an epithelial differentiation marker and a key factor in cell adhesion. EBMD is classified as a corneal dystrophy and can be associated with mutations in corneal keratins, similar to Meesmann dystrophy [6]. In this context, we compared the expression of corneal keratins KRT3, KRT12, and KRT13 in EBMD, SND, and pterygium. The components of retinol and fatty acid metabolism (ADH7, ALDH1A1, FABP5) are altered in conjunctival samples of aniridia patients and potentially involved in the development of aniridia-associated keratopathy and in corneal epithelial changes. Retinoid acid is known to reduce cell adhesion in breast cancer [19] and might also be involved in epithelial alterations in EBMD, SND, or pterygium.

In recent years, the importance of micro RNAs and knowledge about their regulatory function have steadily increased. MicroRNAs (miRNAs) are a class of small non-coding RNAs (~20–24 nucleotides), which regulate most human coding genes at the posttranscriptional level through a specific binding to their target mRNA. Our previous studies revealed significantly altered expression of miR-205-5p in conjunctival impression cytology samples of congenital aniridia subjects [20] and altered miR-138-5p expression in primary LECs of congenital aniridia patients [21].

To systematically investigate whether there are commonalities in gene expression regarding transcription factors PAX6 and TP63, adhesion markers, keratins, markers regarding the retinoic acid metabolism, and microRNAs 138-5p and miR-204-5p, we analyzed gene expression in conjunctival impression cytology samples of patients with EBMD, SND, pterygium, and congenital aniridia and healthy controls and corneal epithelial samples of patients with EBMD, SND, pterygium, and healthy controls.

## 2. Materials and Methods

### 2.1. Sample Collection

Conjunctival impression cytology (IC) and corneal epithelial samples of patients with EBMD, SND, pterygium, congenital aniridia, and healthy controls were collected from patients of the Department of Ophthalmology, Saarland University Medical Center, Homburg/Saar, Germany, during planned corneal surgery. Healthy corneal epithelial samples have been collected at the Department of Ophthalmology, Semmelweis University, Budapest, Hungary, during planned photorefractive keratectomy of healthy individuals. Demographic data of all EBMD, SND, and pterygium patients and healthy individuals are displayed at Table 1.

**Table 1:** Demographic data of patients with Aniridia and healthy subjects.

Control Group			EBMD Group			SND Group			Pterygium Group			Aniridia Group		
IC			IC			IC			IC			IC		
No	Sex	Age (Years)	No	Sex	Age (Years)	No	Sex	Age (Years)	No	Sex	Age (Years)	No	Sex	Age Years
ctr 1	F	32	EBMD 1	F	72	SND 1	F	23	Pt 1	F	44	AN 1	M	14
ctr 2	F	29	EBMD 2	F	64	SND 2	F	59	Pt 2	F	43	AN 2	F	59
ctr 3	M	27	EBMD 3	F	46	SND 3	F	43	Pt 3	F	57	AN 3	M	40
ctr 4	M	15	EBMD 4	F	42	SND 4	M	39	Pt 4	F	59	AN 4	F	57
			EBMD 5	M	40	SND 5	F	63						
**Control Group**			**EBMD Group**			**SND Group**			**Pterygium Group** CEs *					
**CEs**			**CEs**			**CEs**			**(Pooled Samples)**					
**No**	**Sex**	**Age** **(Years)**	**No**	**Sex**	**Age** **(Years)**	**No**	**Sex**	**Age** **(Years)**	**No**	**Sex**	**Age** **(Years)**			
ctr 1	F	33	EBMD 1	F	72	SND 1	F	23	Pt 1 (3)	F	56			
ctr 2	F	40	EBMD 2	F	64	SND 2	F	59	Pt 2 (3)	M	55			
ctr 3	M	22	EBMD 3	F	46	SND 3	F	43	Pt 3 (4)	M	72			
ctr 4	M	18	EBMD 4	F	42	SND 4	M	39	Pt 4 (4)	F	51			
			EBMD 5	M	40	SND 5	F	63						

**Figure 2:**
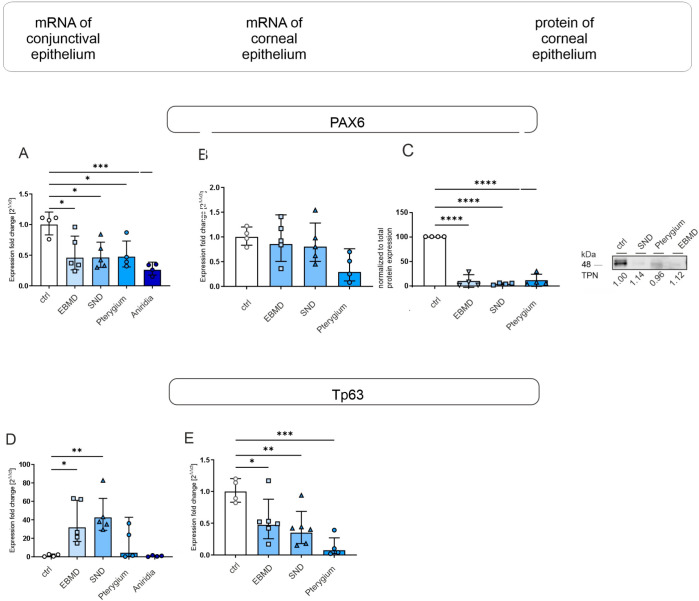
PAX6 and TP63 mRNA expression in conjunctival impression cytology (IC) (**A**,**D**) and corneal epithelial samples (**B**,**E**) and PAX6 protein expression in corneal epithelial samples (**C**) of EBMD, SND, pterygium, and healthy control subjects. Relative levels of mRNA expression were determined by RT-qPCR and protein expression by Western blot analysis. Expression of target genes is presented as geometric mean (RT-qPCR) and arithmetic mean (protein expression) with corresponding standard deviations and a representative Western blot picture. Significances are indicated (* *p* < 0.05, ** *p* < 0.01, *** *p* < 0.001, **** *p* < 0.0001).

* Corneal epithelial samples of patients with pterygium have been pooled from three or four subjects to yield enough protein for WB. The number of subjects included in the pterygium group is in brackets. The described age is the mean age of the subjects (Figure 2 and Table 2).

**Table 2:** Primers used for RT-qPCR of mRNAs and miRNAs.

Name		Cat. No.
PAX6	Paired box gene 6	QT00071169
TP63	Tumor Protein 63	QT02424051
DSG1	Desmoglein-1	QT00001617
OLCN	Occludin	QT00081844
CLDN1	Claudine	QT00225764
CDH1	E-Cadherin	QT00080143
KRT3	Keratin 3	QT00050365
KRT12	Keratin 12	QT00011949
KRT13	Keratin 13	QT00068747
ADH7	Alcohol Dehydrogenase 7	QT00000217
ALDH1A1	Aldehyde Dehydrogenase 1 Family Member A1	QT00013286
FABP5	Fatty Acid Binding Protein 5	QT00225561
GUSB	glucuronidase β	QT00046046
hsa-miR-138-5p		YP00206078
hsa-miR-204-5p		YP00206072
U6 snRNA		YP02119464

Conjunctival impression cytology samples were collected using EYEPRIM™ (Opia, Paris, France) from a conjunctival region beside the pathological corneal changes.

The corneal epithelial samples were collected by corneal abrasion using a hockey knife.

Both conjunctival impression cytology and corneal epithelial samples were directly transferred into lysis buffer provided in the DNA/RNA/Protein Purification Plus Micro Kit (Norgen Biotek CORP., Thorold, ON, Canada) and were stored at −80 °C until RNA and protein extraction.

### 2.2. RNA and Protein Extraction

RNA and proteins were extracted using the DNA/RNA/Protein Purification Plus Micro Kit (Norgen Biotek CORP., Canada), according to the manufacturer’s protocol. For cDNA, 150 ng of total RNA was used as a template for all samples. cDNA was stored at −20 °C until further use.

The protein concentration was analyzed using a commercial Bradford kit using bovine serum albumin as a standard (Merck, Darmstadt, Germany). The absorbance was measured using a microplate reader (TECAN infinite 50, Tecan Deutschland GmbH, Crailsheim, Germany).

### 2.3. Quantitative PCR

For quantitative PCR (qPCR), validated primer sets for use in SYBR Green-based quantitative PCR, obtained from Qiagen GmbH (Hilden, Germany), were utilized (Table 2). The qPCR experiment was carried out for all samples in 96-well plates using AceQ SYBR qPCR Master Mix (Vazyme Biotech, Nanjing, China) and a PCR Thermocycler QuantStudio 5 Real-Time PCR System (ThermoFisher Scientific™ GmbH, Dreieich, Germany). The relative normalized expression of target genes was compared to the respective GUSB reference gene. The ΔΔ cycle threshold (Ct) fold change was quantified by comparing the Ct obtained from the unknown samples compared to the Ct of the reference gene GUSB. For qPCR, the amplification conditions were 95 °C for 10 s, 64 °C for 10 s, and 72 °C for 45 s and 40 cycles.

The miR-204-5p and miR-138-5p expression levels were determined using the miRCURY LNA miRNA RT Kit (Qiagen, Hilden, Germany) and the miRCURY LNA miRNA SYBR Green PCR kit (Qiagen GmbH, Hilden, Germany), and for endogenous control, U6 SnRNA was used. For qPCR, we used the following cycling parameters: 50 °C for 2 min, 95 °C for 2 min, and then 40 cycles of 95 °C for 3 s and 60 °C for 30 s. Results were reported as fold change of the target gene relative to control samples, with all samples normalized to the reference gene following the 2^−ΔΔ Ct^ method.

### 2.4. Western Blot Analysis

To determine PAX6, DSG1, KRT3/76, KRT12, KRT13, ADH7, ALDH1A1, and FABP5 protein expression of corneal epithelial samples, 15 μg protein of the respective cell lysate was used. Detailed information about antibodies is summarized in Table 3. After boiling the samples for 5 min at 95 °C, proteins were separated using NuPAGE ™ bis-tris precast 4–12% bis-tris gels (ThermoFisher Scientific™ GmbH, Dreieich, Germany). Following electrophoresis, the proteins were transferred onto a nitrocellulose membrane with the Trans Blot Turbo Transfer System (BioRad, Hercules, CA, USA). Primary antibodies were diluted in WesternFroxx anti-rabbit or anti-mouse HRP solution containing blocking reagent and secondary antibody (BioFroxx GmbH, Einhausen, Germany). Visualization was performed using an imaging system (iBright, Invitrogen, Waltham, MA, USA). To perform the total protein normalization (TPN), No-Stain™ Protein labeling reagent was purchased from Invitrogen™ (cat. no. A44717). Membranes were incubated in prepared No-Stain labeling solution for 10 min and were imaged using the iBright™ FL1500 Imaging System (Invitrogen, Waltham, MA, USA) to visualize proteins.

### 2.5. Statistical Analysis

Statistical analysis has been performed, and a graphical representation of results was prepared using the GraphPad Prism software (Version 10.2.3). Quantitative PCR data were expressed as geometric mean and geometric mean with SD; Western blot data were expressed as mean ± standard deviation (SD). mRNA expression values (2 ^−∆∆ Ct^ values) and Western blot results were compared to the controls using one-way ANOVA. *p* values <0.05 were considered statistically significant.

## 3. Results

### 3.1. Expression of Transcription Marker PAX6 and TP63, Keratins, Cell-Adhesion Molecules, Components of Retinoic Acid and Fatty Acid Metabolism, and Micro-RNAs in Samples of Patients with EBMD, SND, Pterygium, and Congenital Aniridia

Due to the low protein concentration in IC samples, only mRNA expression could be determined in those using qPCR. The IC samples of patients with EBMD, SND, pterygium, and congenital aniridia were compared to samples of healthy subjects.

#### 3.1.1. Transcription Factors

The transcription factor *PAX6* was significantly reduced at the mRNA level in IC samples from patients with EBMD, SND, pterygium, and congenital aniridia, measuring about half of the levels observed in controls (*p* = 0.016, *p* = 0.012, *p* = 0.02, *p* = 0.001) (Figure 2A). In contrast, TP63 mRNA expression was significantly higher in IC samples from patients with EBMD and SND compared to controls (*p* = 0.019, *p* = 0.004) (Figure 2D).

In corneal epithelial samples from subjects with EBMD, SND, and pterygium, PAX6 mRNA expression did not differ significantly from controls. However, PAX6 protein expression was significantly lower in samples from EBMD, SND, and pterygium patients compared to controls (*p* < 0.0001 for all) (Figure 2B,C). TP63 mRNA expression was significantly lower in SND and pterygium samples than in controls (*p* = 0.042 and *p* = 0.006) (Figure 2E), but TP63 protein could not be detected by Western blot in any of the groups.

#### 3.1.2. Cell Adhesion Molecules

**Figure 3:**
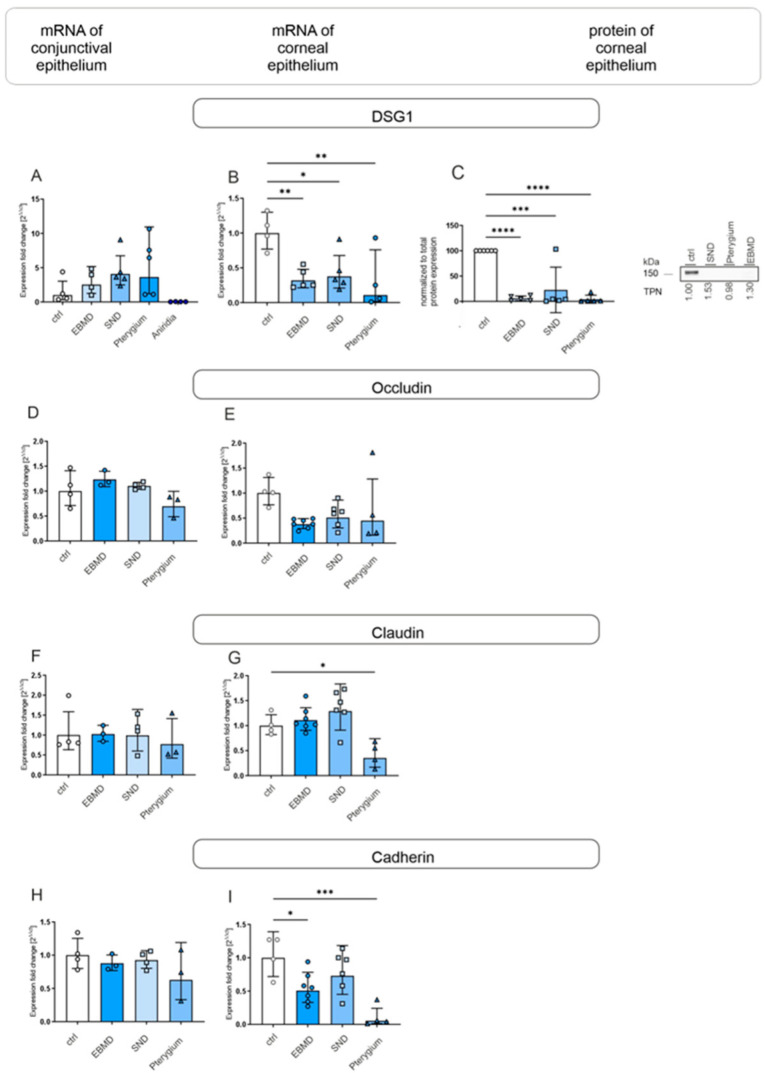
DSG1, occludin, claudin, and cadherin mRNA expression in conjunctival impression cytology (IC) (**A**,**D**,**F**,**H**) and corneal epithelial samples (**B**,**E**,**G**,**I**) and DSG1 protein expression in corneal epithelial samples (**C**) of EBMD, SND, pterygium and healthy control subjects (ctrl). Relative levels of mRNA expression were determined by RT-qPCR and protein expression by Western blot analysis. Expression of target genes is presented as geometric mean (RT-qPCR) and arithmetic mean (protein expression) with corresponding standard deviations and a representative Western blot picture. Significances are indicated (* *p* < 0.05, ** *p* < 0.01, *** *p* < 0.001, **** *p* < 0.0001).

To observe the potential alterations in cell adhesion, we analyzed the adhesion and junction molecules desmoglein 1 (DSG1), occludin (OLCN1), claudin (CLDN1), and cadherin (CDH1). In conjunctival IC samples, no significant changes in mRNA expression were detected in EBMD, SND, or pterygium (Figure 3A,D,F,H). However, both DSG1 mRNA and protein expression were significantly lower in EBMD, SND, and pterygium samples compared to controls (*p* ≤ 0.016 for all) (Figure 3B,C).

Additionally, CLDN1 mRNA expression was significantly reduced in pterygium corneal epithelial samples (*p* = 0.023), and CDH1 mRNA expression was significantly lower in EBMD (*p* = 0.021) and pterygium corneal epithelial samples (*p* = 0.003) than in controls (Figure 3G,I).

#### 3.1.3. Keratins

**Figure 4:**
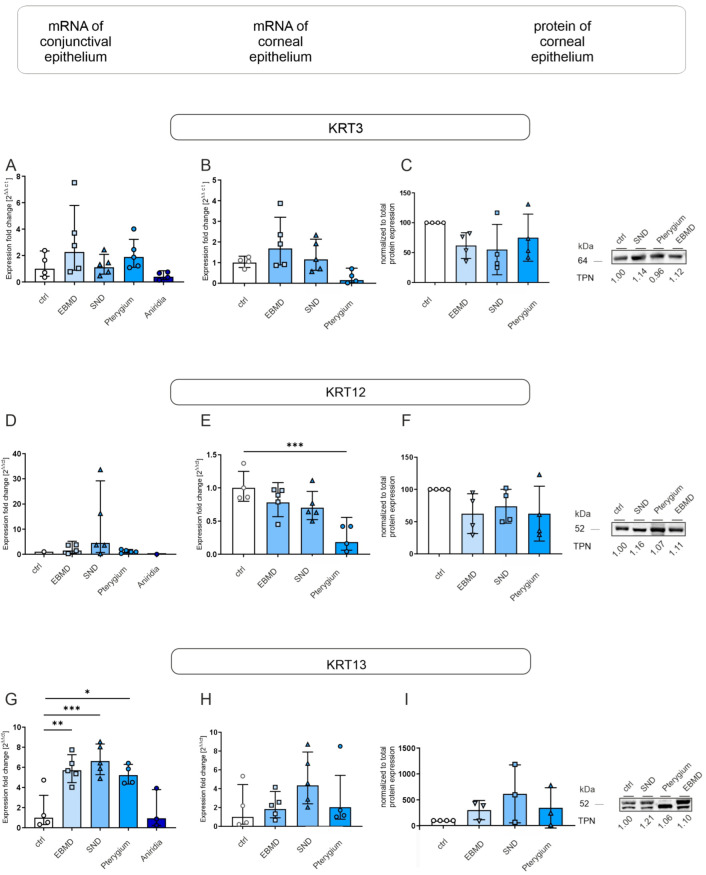
KRT3, KRT12, and KRT13 mRNA expression in conjunctival impression cytology (IC) (**A**,**D**,**G**) and corneal epithelial samples (**B**,**E**,**H**) and KRT3, KRT12, and KRT13 protein expression in corneal epithelial samples (**C**,**F**,**I**) of EBMD, SND, pterygium, and healthy control subjects (ctrl). Relative levels of mRNA expression were determined by RT-qPCR and protein expression by Western blot analysis. Expression of target genes is presented as geometric mean (RT-qPCR) and arithmetic mean (protein expression) with corresponding standard deviations and a representative Western blot picture. Significances are indicated (* *p* < 0.05, ** *p* < 0.01, *** *p* < 0.001).

Since EBMD, SND and pterygium lead to changes in the corneal epithelium, we analyzed levels of the corneal keratins KRT3 and KRT12, as well as the conjunctival keratin 13 (KRT13) in our samples. KRT3, KRT12, and mRNA expression did not differ significantly between any of the groups. KRT13 mRNA expression was significantly higher in EBMD, SND, and pterygium IC samples than in controls (*p* = 0.05; *p* = 0.001; *p* = 0.018) (Figure 4G). FABP5 mRNA expression was significantly higher in pterygium IC samples than in controls (*p* = 0.007) (Figure 4G). KRT12 mRNA expression in corneal epithelial samples was significantly lower in pterygium samples than in controls (*p* = 0.001) (Figure 4E). Nevertheless, KRT3, KRT12, and KRT13 mRNA and protein expression did not differ significantly between any further groups (Figure 4C,F,I).

#### 3.1.4. Molecules of Retinoic Acid and Fatty Acid Metabolism 

**Figure 5:**
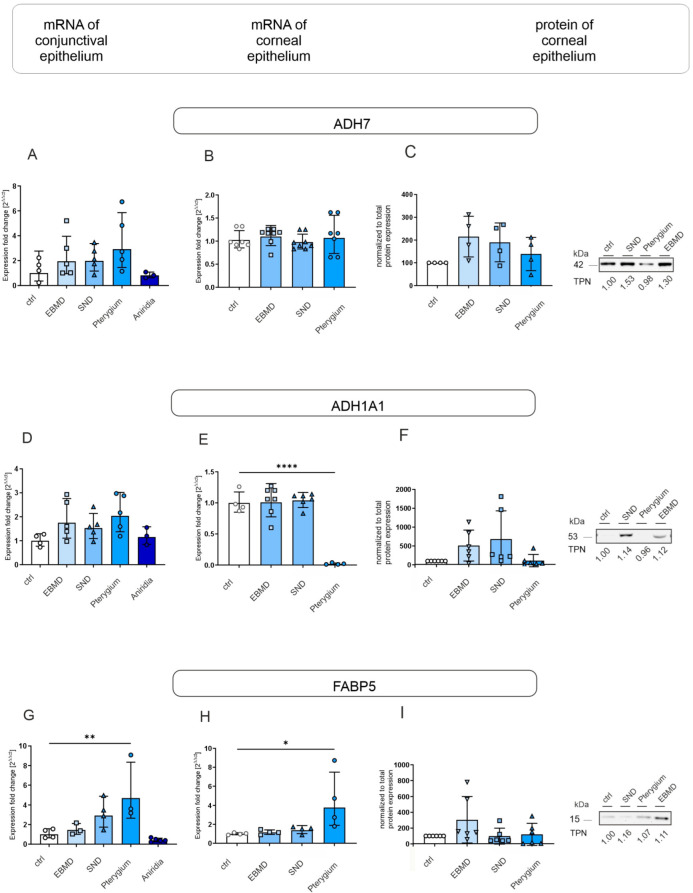
ADH7, ALDH1A1, and FABP5 mRNA expression in conjunctival impression cytology (IC) (**A**,**D**,**G**) and corneal epithelial samples (**B**,**E**,**H**) and ADH7, ALDH1A1, and FABP5 protein expression in corneal epithelial samples (**C**,**F**,**I**) of EBMD, SND, pterygium, congenital aniridia, and healthy control subjects (ctrl). Relative levels of mRNA expression were determined by RT-qPCR and protein expression by Western blot analysis. Expression of target genes is presented as geometric mean (RT-qPCR) and arithmetic mean (protein expression) with corresponding standard deviations and a representative Western blot picture. Significances are indicated (* *p* < 0.05, ** *p* < 0.01, **** *p* < 0.0001).

The alterations in keratins may be a result of the changes in retinoic acid and fatty acid metabolism. ADH7 mRNA and protein expression did not differ significantly between any of the groups (Figure 5A–C). Interestingly, corneal epithelial samples of pterygium subjects showed a decreased ALDH1A1 mRNA expression compared to controls (*p* < 0.0001) (Figure 5E). Nevertheless, its protein expression did not differ significantly between any further groups (Figure 5F). FABP5 mRNA expression was significantly higher in pterygium IC and corneal epithelial samples, than in controls (*p* = 0.007; *p* = 0.014) (Figure 5G,H), whereas its expression remained unchanged in other groups.

#### 3.1.5. Micro-RNAs

**Figure 6:**
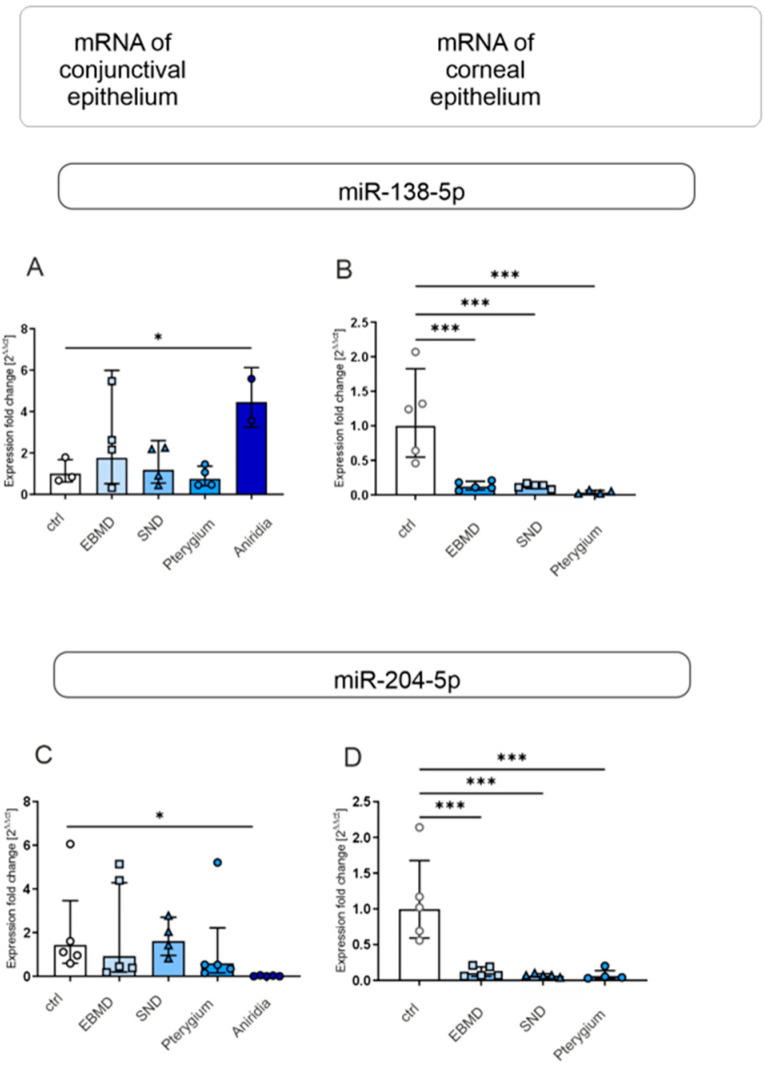
miRNA-138-5p and miR-204-5p expression in conjunctival impression cytology (IC) (**A**,**C**) and corneal epithelial samples (**B**,**D**) of EBMD, SND, pterygium, congenital aniridia, and healthy control subjects (ctrl). Relative levels of miRNA expression were determined by RT-qPCR analysis. Expression of miRNAs is presented as geometric mean (RT-qPCR) with corresponding standard deviations and a representative Western blot picture. Significances are indicated (* *p* < 0.05, *** *p* < 0.001).

MiRNA-138-5p expression was significantly higher, and miRNA 204-5p expression was significantly lower in congenital aniridia than in normal controls (*p* = 0.037; *p* = 0.039) (Figure 6A,C). MiRNA-138 and miR-204-5p mRNA expression was significantly decreased in EBMD, SND, and pterygium epithelial samples compared to controls (*p* = 0.001 for all) (Figure 6B,D). An overview of gene expression of the analyzed markers in EBMD, SND, pterygium, and congenital aniridia in conjunctival IC samples and in corneal epithelial cells is shown in Table 4.

## 4. Discussion

In the present study, we compared stem cell and adhesion markers, keratins, markers related to retinoic acid and fatty acid metabolism, and micro-RNAs in conjunctival and corneal epithelial cells of patients with EBMD, SND, pterygium, and congenital aniridia with healthy control samples. We aimed to compare these results with already described deregulated markers in congenital aniridia epithelial cells, which are also characterized by limbal stem cell deficiency.

The most important result of the current study is a decreased PAX6 mRNA expression in EBMD, SND, and pterygium conjunctival IC samples and a decreased PAX6 protein expression in EBMD, SND, and pterygium corneal epithelial cells. Additionally, we could detect a decreased DSG1 mRNA expression in EBMD, SND, and pterygium in corneal epithelial cells and a correspondingly decreased protein expression. Interestingly, our measurement results in EBMD, SND, and pterygium in central corneal epithelial cells show similarities with limbal epithelial cells in AAK, particularly PAX6 and DSG1 mRNA and protein expression [22,23,24]. In the corneal epithelium, we found a decreased PAX6 protein expression and decreased DSG1 mRNA and protein expression in EBMD, SND, and pterygium samples. We know from previous studies that reduced DSG1 expression correlates with reduced PAX6 expression [23] and that DSG1 is most probably a downstream target of PAX6.

TP63 is a transcriptional regulator essential for the migration and proliferation of corneal epithelial cells. It is highly expressed in limbal epithelial stem cells, where it helps to maintain a high proliferative potential [25]. Elevated TP63 expression has been observed in conjunctival intraepithelial neoplasia (CIN) compared to healthy controls [26]. The results of our study indicate that TP63 mRNA expression is increased in EBMD and SND conjunctival impression cytology (IC) samples, likely reflecting an ocular surface disease characterized by elevated proliferation and inflammation in these patients. Additionally, the variability in TP63 mRNA expression across EBMD, SND, and pterygium IC samples suggests increased disease activity.

Nevertheless, TP63 mRNA expression was decreased in corneal epithelial cells of both SND and pterygium subjects, referring to a slower turnover of these epithelial cells.

A further assumption of our group was that the corneal epithelium in EBMD, SND, and pterygium could reveal alterations in cell adhesion and tight junctions. We analyzed DSG1, which is a differentiation marker and also essential for intercellular adhesion. In addition, there is an increased proliferation of corneal epithelial cells when their expression is reduced. DSG1 is responsible for the maintenance of the epithelial barrier function, and therefore, reduced DSG1 levels could result in a reduced corneal epithelial cell adhesion [27,28].

The current results, which show DSG1 mRNA and protein expression decrease in EBMD, SND, and pterygium, correlate well with the clinical observation that EBMD, SND, and pterygium subjects suffer from recurrent corneal erosions [5,7]. Interestingly, expression of the tight junction molecule occludin expression did not differ between the observed groups. Claudin mRNA expression was decreased in corneal epithelial samples of pterygium patients, and E-cadherin mRNA expression was decreased in the epithelium of EBMD and pterygium subjects. The authors suggest that the differentiation of the corneal epithelium is more affected than alterations in cell adhesion in the analyzed disease entities.

Among keratins, a significantly increased KRT13 mRNA expression in conjunctival IC samples was noticeable in EBMD, SND, and pterygium patients. KRT13 is a conjunctival epithelial cell marker [29]. The increased TP63 expression with a simultaneously increased KRT13 expression in conjunctival IC samples of EMBD, SND, and pterygium patients suggests an increased activity of conjunctival stem cells leading to an increased conjunctival epithelial cell production, maybe also related to ocular surface inflammation [30,31].

KRT12 and KRT3 are both proteins that are expressed in the corneal epithelium. KRT12 mRNA expression was decreased in corneal epithelial cells of our pterygium samples; nevertheless, KRT3 expression did not differ between any analyzed groups. Similar results have already been described by Jaworski et al. in 2009 [13].

ADH7 and ADH1A1 are important for corneal epithelial cell maintenance [22,23,32]. In congenital aniridia, with PAX6 haploinsufficiency, we could observe deregulated expression of the retinoic acid signaling components ADH7 and ADH1A1 in previous studies [22,23,32]. A decreased FABP5 and DSG1 mRNA expression was also detected following PAX6 knockdown in human limbal epithelial cells, using an aniridia cell model [23]. Nevertheless, ADH7 expression changes were not observed in any of our patient groups, and ADH1A1 expression was solely deregulated in epithelial cells of pterygium patients at the transcriptional level, but not in any other groups. In addition, interestingly, there was an increased FABP5 mRNA expression without an increased FABP5 protein expression, parallel to a decreased PAX6 and DSG1 expression in conjunctival IC samples and in corneal epithelium of pterygium patients. These data refer to deregulated retinoic acid metabolism of the ocular surface of pterygium subjects, but not in EBMD and SND. Nevertheless, our data contradict our previous measurement results in epithelial cells using an aniridia cell model, where a decreased FABP5 and DSG1 level could be verified, parallel to a decreased PAX6 expression [23]. The exact molecular mechanisms behind this phenomenon still need to be clarified.

In recent years, the importance of micro RNAs and knowledge about their regulatory influence have steadily increased. MicroRNAs (miRNAs) are a class of small non-coding RNAs (∼20–24 nucleotides), which regulate most human coding genes at the posttranscriptional level through a specific binding to their target mRNA and are involved in the development of cardiovascular diseases and cancer [33,34].

Our previous studies revealed significantly altered expression of miR-204-5p in conjunctival impression cytology samples of congenital aniridia subjects [20] and altered miR-138-5p expression in primary LECs of congenital aniridia patients (*p* = 0.03) [21]. Therefore, it was very interesting for us to observe the decreased expression of miR-138-5p and mirR-204-5p in corneal epithelial cells of EBMD, SND, and pterygium subjects, which may indicate an increasing cell growth and migration in these subjects [35,36,37]. It is known that the downregulation of these micro-RNAs is found in many cancers. In corneal wound healing studies, using human corneal epithelial cell lines, the transfection of miRNA-204-5p resulted in decreased cell proliferation and migration [36]. Since there is a known co-regulation of PAX6 and miR-204-5p in eye development, it is conceivable that this co-regulation also exists in the corneal epithelium [38].

These findings also support the hypothesis that in the pathomechanism of EBMD, SND and pterygium, there is a loss of epithelial cell function, which is at many points similar to that in aniridia associated keratopathy. Nevertheless, inflammation may also play an important role in disease development. A better understanding of the molecular mechanisms could support us with alternative treatment options for these ocular surface diseases.

## 5. Conclusions

PAX6, DSG1, miR-138-5p, and miR-204-5p expression is decreased in the corneal epithelium of epithelial basal membrane dystrophy, Salzmann nodular degeneration, and pterygium subjects. In addition, there is a deregulation of markers of the retinoic acid signaling pathway, such as ADH1A1 and FABP5 in the corneal epithelium of pterygium subjects. These changes may offer therapeutic targets in the treatment of these ocular surface diseases.

## Figures and Tables

**Table 3:** Primary antibodies used for Western blot.

Antibody	Dilution	Cat. No.	Manufacturer
PAX6 rabbit polyclonal	1:1000	AB 2237	Merck Millipore, Watford, UK
DSG1 mouse monoclonal	1:1000	Sc-59904	Santa Cruz Biotechnology, Dallas, TX, USA
Keratin 3/76 mouse monoclonal	1:2000	CBL218	Merck Millipore, Watford, UK
Keratin 12 mouse monoclonal	1:1000	Sc-515882	Santa Cruz Biotechnology, Dallas, TX, USA
Keratin 13 mouse monoclonal	1:1000	Sc-101460	Santa Cruz Biotechnology, Dallas, TX, USA
ADH 7 rabbit polyclonal	1:1000	PA5-98484	Thermo Fisher Scientific, Waltham, MA, USA
ALDH1A1mouse monoclonal	1:1000	Sc-374076	Santa Cruz Biotechnology, Dallas, TX, USA
FABP5 rabbit polyclonal	1:1000	12348-1-AP	Proteintech, Planegg-Martinsried, Germany

**Table 4:** Overview of gene and protein expression in epithelial basal membrane dystrophy, Salzmann’s nodular degeneration, and pterygium.

	Conjunctival Epithelium mRNA				Corneal Epithelium mRNA			Corneal Epithelium Protein		
	EBMD	SND	Ptery- gium	Aniridia	EBMD	SND	Ptery- gium	EBMD	SND	Ptery- gium
PAX6	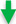	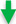	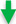	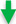	n.d.	n.d.	n.d.	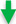	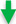	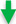
Tp63	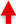	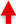	n.d.	n.d.	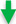	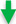	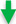	n.a.	n.a.	n.a.
DSG1	n.d.	n.d.	n.d.	n.d	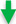	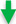	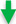	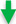	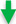	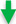
Occludine	n.d.	n.d.	n.d.	n.d.	n.d.	n.d.	n.d.	n.a.	n.a.	n.a.
Claudine	n.d.	n.d.	n.d.	n.a.	n.d.	n.d.	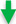	n.a.	n.a.	n.a.
E-Cadherin	n.d.	n.d.	n.d.	n.a.	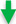	n.d.	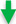	n.a.	n.a.	n.a.
KRT3	n.d.	n.d.	n.d.	n.d.	n.d.	n.d.	n.d.	n.d.	n.d.	n.d.
KRT12	n.d.	n.d.	n.d.	n.d.	n.d.	n.d.	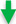	n.d.	n.d.	n.d.
KRT13	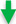	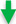	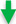	n.d.	n.d.	n.d.	n.d.	n.d.	n.d.	n.d.
ADH7	n.d.	n.d.	n.d.	n.d.	n.d.	n.d.	n.d.	n.d.	n.d.	n.d.
ALDH1A1	n.d.	n.d.	n.d.	n.d.	n.d.	n.d.	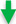	n.d.	n.d.	n.d.
FABP5	n.d.	n.d.	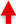	n.d.	n.d.	n.d.	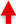	n.d.	n.d.	n.d.
miR-138-5p	n.d.	n.d.	n.d.	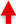	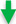	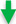	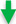	n.a.	n.a.	n.a.
miR-204-5p	n.d.	n.d.	n.d.	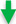	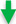	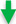	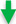	n.a	n.a.	n.a.

n.a. = not applicable. n.d. = no difference compared to the control. Green arrows = downregulatd mRNA or protein expression, red arrows = upregulated mRNA or protein expression compared to the control.

## Data Availability

All data generated or analyzed during this study are included in this article. Further inquiries can be directed to the corresponding author.

## References

[1] Peng J., Sha X.Y., Liu Y., Yang R.M., Wen Y. (2015). Pterygium epithelium abnormal differentiation related to activation of extracellular signal-regulated kinase signaling pathway in vitro. Int. J. Ophthalmol..

[2] Paranjpe V., Galor A., Monsalve P., Dubovy S.R., Karp C.L. (2019). Salzmann nodular degeneration: Prevalence, impact, and management strategies. Clin. Ophthalmol..

[3] Jadczyk-Sorek K., Garczorz W., Bubała-Stachowicz B., Francuz T., Mrukwa-Kominek E. (2023). Matrix Metalloproteinases and the Pathogenesis of Recurrent Corneal Erosions and Epithelial Basement Membrane Dystrophy. Biology.

[4] Bausili M.M., Alvarez De Toledo J., Barraquer R.I., Michael R., Tresserra F., De La Paz M.F. (2016). Histopathology Findings of Corneal Buttons in Congenital Aniridia Patients. Ophthalmic Res..

[5] Pham L.T.L., Goins K.M., Sutpin J.E., Wagoner M.D. Treatment of Epithelial Basement Membrane Dystrophy with Manual Superficial Keratectomy. EyeRounds.org. http://webeye.ophth.uiowa.edu/eyeforum/cases/78-EBMD-treatment.htm.

[6] Weiss J.S., Rapuano C.J., Seitz B., Busin M., Kivelä T.T., Bouheraoua N., Bredrup C., Nischal K.K., Chawla H., Borderie V. (2024). IC3D Classification of Corneal Dystrophies—Edition 3. Cornea.

[7] Maharana P.K., Sharma N., Das S., Agarwal T., Sen S., Prakash G., Vajpayee R.B. (2016). Salzmann’s Nodular Degeneration. Ocul. Surf..

[8] Hamada S., Darrad K., McDonnell P.J. (2011). Salzmann’s nodular corneal degeneration (SNCD): Clinical findings, risk factors, prognosis and the role of previous contact lens wear. Contact Lens Anterior Eye.

[9] Farjo A.A., Halperin G.I., Syed N., Sutphin J.E., Wagoner M.D. (2006). Salzmann’s nodular corneal degeneration clinical characteristics and surgical outcomes. Cornea.

[10] Eberwein P., Hiss S., Auw-Haedrich C., Sundmacher R., Hauer K., Boehringer D., Meier P., Reinhard T. (2010). Epithelial marker expression in Salzmann nodular degeneration shows characteristics of limbal transient amplifying cells and alludes to an involvement of the epithelium in its pathogenesis. Acta Ophthalmol..

[11] Suarez M.F., Echenique J., López J.M., Medina E., Irós M., Serra H.M., Fini M.E. (2021). Transcriptome Analysis of Pterygium and Pinguecula Reveals Evidence of Genomic Instability Associated with Chronic Inflammation. Int. J. Mol. Sci..

[12] Xu N., Cui Y., Dong J., Huang L. (2020). Exploring the Molecular Mechanisms of Pterygium by Constructing lncRNA-miRNA-mRNA Regulatory Network. Investig. Ophthalmol. Vis. Sci..

[13] Jaworski C.J., Aryankalayil-John M., Campos M.M., Fariss R.N., Rowsey J., Agarwalla N., Reid T.W., Dushku N., Cox C.A., Carper D. (2009). Expression analysis of human pterygium shows a predominance of conjunctival and limbal markers and genes associated with cell migration. Mol. Vis..

[14] de Almeida Borges D., Alborghetti M.R., Franco Paes Leme A., Ramos Domingues R., Duarte B., Veiga M., Trindade Ferrer M., Viana Wanzeler A.C., Leite Arieta C.E., Alves M. (2020). Tear proteomic profile in three distinct ocular surface diseases: Keratoconus, pterygium, and dry eye related to graft-versus-host disease. Clin. Proteom..

[15] Winiarczyk M., Biela K., Michalak K., Winiarczyk D., Mackiewicz J. (2022). Changes in Tear Proteomic Profile in Ocular Diseases. Int. J. Environ. Res. Public Health.

[16] Rezvan F., Khabazkhoob M., Hooshmand E., Yekta A., Saatchi M., Hashemi H. (2018). Prevalence and risk factors of pterygium: A systematic review and meta-analysis. Surv. Ophthalmol..

[17] Secker A., Daniels J.T. (2009). Limbal Epithelial Stem Cells of the Cornea.

[18] He S., Wu Z. (2022). Biomarkers in the Occurrence and Development of Pterygium. Ophthalmic Res..

[19] Castro-Guijarro A.C., Vanderhoeven F., Mondaca J.M., Redondo A.L., Zoppino F.C.M., Fernandez-Muñoz J.M., Sanchez A.M., Flamini M.I. (2022). Combination Treatment of Retinoic Acid Plus Focal Adhesion Kinase Inhibitor Prevents Tumor Growth and Breast Cancer Cell Metastasis. Cells.

[20] Latta L., Ludwig N., Krammes L., Stachon T., Fries F.N., Mukwaya A., Szentmáry N., Seitz B., Wowra B., Kahraman M. (2021). Abnormal neovascular and proliferative conjunctival phenotype in limbal stem cell deficiency is associated with altered microRNA and gene expression modulated by PAX6 mutational status in congenital aniridia. Ocul. Surf..

[21] Nastaranpour M., Suiwal S., Stachon T., Fries F.N., Amini M., Seitz B., Meese E., Ludwig N., Szentmáry N. (2025). miRNA Expression Profile in Primary Limbal Epithelial Cells of Aniridia Patients. Investig. Ophthalmol. Vis. Sci..

[22] Latta L., Nordstrom K., Stachon T., Langenbucher A., Fries F.N., Szentmary N., Seitz B., Kasmann-Kellner B. (2019). Expression of retinoic acid signaling components ADH7 and ALDH1A1 is reduced in aniridia limbal epithelial cells and a siRNA primary cell based aniridia model. Exp. Eye Res..

[23] Katiyar P., Stachon T., Fries F.N., Parow F., Ulrich M., Langenbucher A., Cayless A., Seitz B., Käsmann-Kellner B., Latta L. (2021). Decreased FABP5 and DSG1 protein expression following PAX6 knockdown of differentiated human limbal epithelial cells. Exp. Eye Res..

[24] Roux L.N., Petit I., Domart R., Concordet J.P., Qu J., Zhou H., Joliot A., Ferrigno O., Aberdam D. (2018). Modeling of Aniridia-Related Keratopathy by CRISPR/Cas9 Genome Editing of Human Limbal Epithelial Cells and Rescue by Recombinant PAX6 Protein. Stem Cells.

[25] Di Iorio E., Barbaro V., Ruzza A., Ponzin D., Pellegrini G., De Luca M. (2005). Isoforms of DeltaNp63 and the migration of ocular limbal cells in human corneal regeneration. Proc. Natl. Acad. Sci. USA.

[26] Auw-Haedrich C., Sundmacher R., Freudenberg N., Spelsberg H., Feltgen N., Maier P., Reinhard T. (2006). Expression of p63 in conjunctival intraepithelial neoplasia and squamous cell carcinoma. Graefe’s Arch. Clin. Exp. Ophthalmol..

[27] Sherrill J.D., Kc K., Wu D., Djukic Z., Caldwell J.M., Stucke E.M., Kemme K.A., Costello M.S., Mingler M.K., Blanchard C. (2014). Desmoglein-1 regulates esophageal epithelial barrier function and immune responses in eosinophilic esophagitis. Mucosal Immunol..

[28] Polivka L., Hadj-rabia S., Bal E., Madrange M., Hamel Y., Bonnet D., Lepidi H., Ovaert C., Barbet P., Dupont C. (2018). Epithelial barrier dysfunction in desmoglein-1 deficiency. J. Allergy Clin. Immunol..

[29] Ramirirez-Miranda A., Nakatsu M., Zarei-Ghanavati S., Nguyen C., Deng S. (2011). Keratin 13 is a more specific marker of conjunctival epithelium than keratin 19. Mol. Vis..

[30] Cumplido-Laso G., Benitez D.A., Mulero-Navarro S., Carvajal-Gonzalez J.M. (2023). Transcriptional Regulation of Airway Epithelial Cell Differentiation: Insights into the Notch Pathway and Beyond. Int. J. Mol. Sci..

[31] Zhong Z., Chen S. (2022). Isolation and Expansion of Primary Conjunctival Stem Cells (CjSCs) from Human and Rabbit Tissues. Bio Protoc..

[32] Latta L., Knebel I., Bleil C., Stachon T., Katiyar P., Zussy C., Fries F.N., Käsmann-Kellner B., Seitz B., Szentmáry N. (2021). Similarities in DSG1 and KRT3 Downregulation through Retinoic Acid Treatment and PAX6 Knockdown Related Expression Profiles: Does PAX6 Affect RA Signaling in Limbal Epithelial Cells?. Biomolecules.

[33] Chakrabortty A., Patton D.J., Smith B.F., Agarwal P. (2023). miRNAs: Potential as Biomarkers and Therapeutic Targets for Cancer. Genes.

[34] Li H., Zhan J., Chen C., Wang D. (2022). MicroRNAs in cardiovascular diseases. Med. Rev..

[35] Yeh M., Wang Y.Y., Yoo J.Y., Oh C., Otani Y., Kang J.M., Park E.S., Kim E., Chung S., Jeon Y.J. (2021). MicroRNA-138 suppresses glioblastoma proliferation through downregulation of CD44. Sci. Rep..

[36] An J., Chen X., Chen W., Liang R., Reinach P.S., Yan D., Tu L.L. (2015). MicroRNA expression profile and the role of miR-204 in corneal wound healing. Investig. Ophthalmol. Vis. Sci..

[37] Eskildsen T., Taipaleenmäki H., Stenvang J., Abdallah B.M., Ditzel N., Nossent A.Y., Bak M., Kauppinen S., Kassem M. (2011). *MicroRNA*-138 regulates osteogenic differentiation of human stromal (mesenchymal) stem cells in vivo. Proc. Natl. Acad. Sci. USA.

[38] Shaham O., Gueta K., Mor E., Oren-Giladi P., Grinberg D., Xie Q., Cvekl A., Shomron N., Davis N., Keydar-Prizant M. (2013). Pax6 Regulates Gene Expression in the Vertebrate Lens through miR-204. PLoS Genet..

